# Self-monitoring of Oral Health Using Smartphone Selfie Powered by Artificial Intelligence: Implications for Preventive Dentistry

**DOI:** 10.3290/j.ohpd.5758200

**Published:** 2024-07-23

**Authors:** Reinhard Chun-Wang Chau, Khaing Myat Thu, Richard Tai-Chiu Hsung, Colman McGrath, Walter Yu-Hang Lam

**Affiliations:** a PhD Candidate, Faculty of Dentistry, The University of Hong Kong, Hong Kong, China. Idea, hypothesis, experimental design, wrote the manuscript, contributed substantially to discussion.; b Senior Research Assistant, Faculty of Dentistry, The University of Hong Kong, Hong Kong, China. Idea, hypothesis, experimental design, wrote the manuscript, contributed substantially to discussion.; c Associate Professor, Department of Computer Science, Hong Kong Chu Hai College, Hong Kong, China. Idea, proofread the manuscript, contributed substantially to discussion.; d Clinical Professor in Dental Public Health and Division Coordinator, Faculty of Dentistry, The University of Hong Kong, Hong Kong, China. Proofread the manuscript, contributed substantially to discussion.; e Clinical Assistant Professor in Prosthodontics, Faculty of Dentistry, The University of Hong Kong, Hong Kong; Member, Musketeers Foundation Institute of Data Science, The University of Hong Kong, Hong Kong, China. Idea, hypothesis, experimental design, proofread the manuscript, contributed substantially to discussion.; *Both authors contributed equally, names in alphabetical order.

**Keywords:** artificial intelligence, dental, health education, oral health, precision dentistry, precision medicine

## Abstract

**Purpose:**

With the increasing use of artificial intelligence (AI) in dentistry, it is feasible to self-monitor oral health using Oral Health AI Advisors (OHAI Advisors). This technological advancement offers the potential for early detection of oral diseases and facilitates early prevention. This systematic review aimed to evaluate the effectiveness of OHAI Advisors as a tool in preventive dentistry for the general population.

**Materials and Methods:**

Standardised searches were performed and screened across four electronic databases. The primary outcomes were changes in clinical and behavioural measures, and evidence was synthesised. The quality of the included studies was assessed.

**Results:**

The initial search identified 1639 articles, 64 full texts were reviewed, and four studies were included in the analyses. Qualitative synthesis revealed that short-term use of OHAI Advisors, for up to 6 months, statistically significantly reduced plaque and gingival index scores. Combining OHAI Advisors with verbal counseling enhanced their effectiveness. No studies investigated effects on oral health awareness, behavioural changes, or adherence to regular practice. The risk of bias in the included studies was moderate to low.

**Conclusion:**

OHAI Advisors appear to be effective for short-term oral hygiene maintenance. Further research is necessary to determine the preventive capability, focusing on assessing long-term outcomes on oral health and any changes in oral health behaviour.

The global oral health burden is an urgent public-health challenge. Many people face difficulties accessing dental care for disease detection and to obtain preventive advice.^7^ Unfavourable dentist:patient ratios, socioeconomic factors, and financial costs are significant barriers to accessing oral health care and preventive services.^23^ Irregular dental visits result in missed opportunities for early detection and prevention of oral disease,^2^ often resulting in more costly and complex dental treatment for rehabilitation.^19^ It is highly important to implement and monitor effective self-care measures for plaque control, such as toothbrushing and interdental cleaning,^14^ as they prevent or control plaque-induced diseases.^3^

Oral Health Instruction (OHI) has long been a key preventive strategy to maintain optimal oral hygiene and prevent disease.^31^ Traditionally, this has been through direct patient education (1-on-1), although its cost-effectiveness has been challenged.^30^ Nonetheless, patients have expressed a preference for more personalised OHI from dental professionals, also to monitor outcomes.^4^ To this end, there exist alternative modes of delivering oral health preventive advice.^22^^,^^29^

Mobile health (mHealth) technology is increasingly proposed to accurately detect diseases^1^ and potentially provide personalised feedback and advice. mHealth has also been adopted in oral healthcare, improving adherence and compliance with OHI.^17^ Additionally, the development of an artificial intelligence (AI) network has facilitated the automatic evaluation of intraoral photographs for dental plaque and gingival inflammation with high accuracy.^3^ In recent years, integrating AI into smartphones as mHealth applications has considerably improved oral health monitoring and management.^1^^,^^17^

Globally, smartphones with high-quality cameras have increased to facilitate mHealth through image capture.^28^ AI algorithms can analyse these images using advanced feature extraction, pattern recognition, and machine learning techniques.^5^^,^^12^ Remote assessment offers great potential for accurately detecting and diagnosing oral health conditions, notably early-stage periodontal diseases like gingivitis.^8^ Combining AI-powered systems with smartphone technology enables remote consultations and personalised feedback to promote behavioural changes and adherence to oral hygiene practices.^4^^,^^10^

This review aimed to determine the use of OHAI Advisors and their effectiveness in clinical and behavioural outcomes. This review’s findings support the use of OHAI Advisor as a tool of preventive dentistry and identify areas for further development and translation into oral health promotion practices.

## MATERIALS AND METHODS

This review followed the Preferred Reporting Items for Systematic Reviews and Meta-analyses (PRISMA) statement.^18^

### Review Question and Criteria

The Population, Intervention, Control, and Outcomes (PICO) framework was employed to answer the question, “Does the application of Oral Health AI Advisor (OHAI Advisor) for self-monitoring oral health effectively improve clinical and behavioural outcomes?” The population of interest (P) was the general population. The intervention was OHAI Advisor via a digital platform or application using intraoral or dental selfies from a smartphone (I). In contrast, conventional face-to-face oral hygiene instruction was the comparator (C). Both clinical and behavioural changes were considered as the outcomes (O).

Both randomised and non-randomised control trials were included in this review. The detailed selection criteria are delineated in Table 1.

**Table 1:** Inclusion/exclusion criteria of this review

Search term set (#1) Interventions	[(“selfie) OR (photograph) OR (camera) OR (smartphone) OR (“mobile application”)]
Search term set (#2) Population	[(“dental plaque”) OR (“dental caries”) OR (“dental biofilm”) OR (“dental hygiene”) OR (“oral hygiene”) OR (gum) OR (gingiva) OR (gingivitis) OR (periodontal)]
Filters	Humans, English, Adult: 19+ years, Young Adult: 19-24 years, Adult: 19-44 years, Middle Aged + Aged: 45+ years, Middle Aged: 45-64 years, Aged: 65+ years, 80 and over: 80+ years, Exclude preprints, from 1990 - 2023
Search combination	(#1) AND (#2)
Inclusion criteria	Original clinical studies on human Adolescent to older adult Not specific to the type of subjects (pregnant, medically compromised, etc.) Studies with oral health monitoring in which the AI system receives the selfie photographs, evaluates, and responds to the users for necessary information Must be using smartphone photography (rear camera or selfie) to take intraoral/teeth/mouth photo Studies analyse oral hygiene or dental plaque, dental caries, or gum diseases
Exclusion criteria	Studies without statistical analysis Studies that did not use smartphone selfies in OHAI Advisors Studies that focused on the perspectives of healthcare workers instead of patients Clinical or professional photography, intraoral scanner, or other imaging Professional diagnosis of an oral disease other than oral hygiene, dental plaque, dental caries, or gum diseases (such as cancer, white or red colour changes, etc.) Intraoral photographs analysed and diagnosed by dental professional personnel

### Search Strategy

Two assessors independently searched a literature search across four electronic databases: PubMed, Scopus, Cochrane Library, and Web of Science. Additional searches were conducted via Google Scholar, ResearchGate, and reference snowballing of included studies from inception until August 2023. Only studies written in English and featuring human clinical cohorts were included. Reviews, case reports, commentaries, study protocols, and studies without statistical analysis were excluded.

### Study Selection

Duplication checks were performed on the studies, and title and abstract screening was independently conducted by two assessors (R.C.W.C., K.M.T.) using an online platform (Covidence).^11^ Disagreements were resolved through discussion. The two assessors then independently reviewed full-text articles to select eligible studies using the same online platform, with any conflicts again resolved by discussion.

### Data Extraction

Data extraction was independently conducted by two researchers using the aforementioned online platform. The risk of bias was assessed independently using the National Institutes of Health (NIH) study quality assessment tools.^15^^,^^26^ The evaluation of domains of group allocation, including randomisation and blinding, drop-out rate, specification of intervention and outcome, and sample size calculation, was performed to determine study quality. The following study features were extracted: application of the OHAI Advisor systems; features of the applied OHAI Advisor systems; reported outcomes. A third assessor (W.Y.H.L.) reviewed the extracted data to ensure quality. The corresponding authors of studies with missing details in the publication were contacted.

## RESULTS

### Search Results

**Fig 1:**
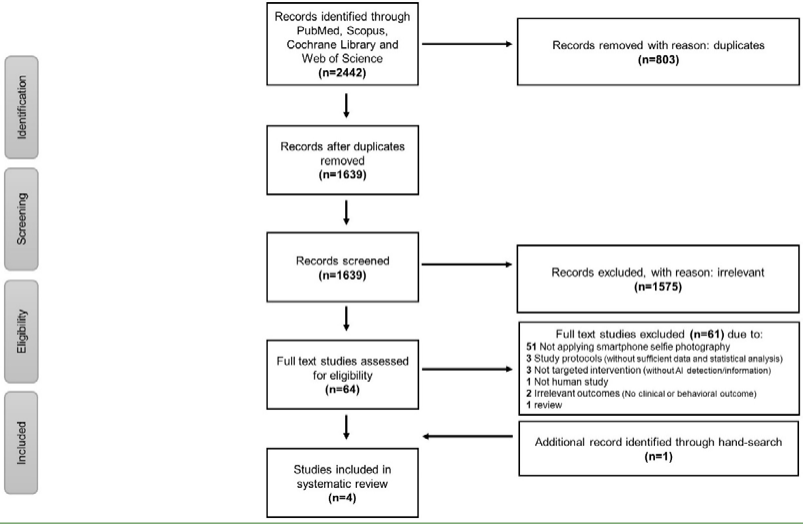
PRISMA flowchart of this review.

The primary literature search yielded a total of 2442 studies. After removing duplicates, 1639 studies were screened. Based on screening titles and abstracts, 64 studies were shortlisted for inclusion. The full text of the shortlisted studies underwent an eligibility assessment, resulting in the selection of three studies. An additional study was manually retrieved from the selected articles’ reference list, meeting the targeted intervention criteria. The final count of studies included in this review was 4 (Fig 1).

### Study Characteristics

The selected studies (n = 4)^20^^,^^21^^,^^24^^,^^25^ encompassed 264 participants (Table 2). Among them, 137 were assigned to receive interventions with an OHAI Advisor. In contrast, 127 were assigned to control groups where face-to-face OHI was used. All four selected studies utilised OHAI Advisor, with three^20^^,^^24^^,^^25^ employing the Dental Monitoring application (DentalMonitoring, Paris, France) and one^21^ using the WhiteTeeth application (University of Amsterdam, Amsterdam, Netherlands). Participants in three studies^20^^,^^24^^,^^25^ were recruited from university clinics or hospitals: the University of Illinois Chicago’s orthodontic clinic,^25^ the teaching hospital of the University of Brescia (Italy),^20^ and the Division of Periodontics of Kaohsiung Medical University (Taiwan).^24^ In contrast, the remaining study^21^ recruited participants from orthodontic clinics in Alkmaar and Leiden (the Netherlands). Three studies^20^^,^^21^^,^^25^ focused on orthodontic patients, and one^24^ targeted periodontal treatments in the adult population. OHAI Advisor was administered weekly in three studies.^21^^,^^24^^,^^25^ Among them, two studies^21^^,^^24^ followed up on outcomes for three months and one^25^ for 13 months. The remaining study^20^ adopted a monthly OHAI Advisor regimen and conducted six-month assessments.

**Table 2:** The summary of included studies

Study (in ­alphabetical order)	Study Design	Characteristics of samples	Sample size of the study and control group	Intervention	Smartphone intraoral photographs were taken by whom	Parameters investigated by smartphone photographs	Reminder to adhere to the practice	Assessments
**Sangalli et al^20^**	NRCT	Patients receiving orthodontic treatments (mean age: 20.6 yrs; mean age for male: 14.0 yrs; mean age for female:16 years)	Total (n) = 30 Study group (n=15) Control group (n=15)	Study group: attended conventional chair-side appointments and instructed to use DM scans monthly, with the app sending text messages to the patient Control group: attended conventional chair-side appointments	By subjects themselves with a rear camera, a scan box, and cheek retractors	Non-disclosed dental plaque Periodontal gingival health	No reminder	Plaque index Modified gingival index Number of white spot lesions
**Scheerman et al^21^**	RCT	Adolescent patients receiving orthodontic treatment (mean age for study group: 13.2 yrs; mean age for control group:13.5 years)	Total (n) = 132 Study group (n=65) Control group (n=67)	Study group: used the WhiteTeeth app weekly for 12 weeks and received tailored feedback from AI Control group: conventional oral health education and oral health instruction by dental-care providers once after each appointment	Selfie photographs with front camera by patients	Disclosed dental plaque	Reminded by the app automatically once per week	Al-Anezi and Harradine plaque index Bleeding on Marginal Probing Index Oral health behaviour score Tooth brushing frequency and duration Use of interproximal brush use Use of fluoride mouth rinse
**Shen et al^24^**	RCT	Adult patients with periodontitis (mean age: 45.0 yrs)	Total (n) = 53 Study group 1 (n=16) Study group 2 (n=17) Control group (n=25)	Study group 1: conventional oral health instruction by a dental hygienist post appointment, and instructed to use the DM app for three months and receive canned messages from the app Study group 2: conventional oral health instruction by a dental hygienist post appointment and instructed to use the DM app for three months and receive canned messages from the app, in addition to individualised oral health counseling by another dental hygienist Control group: conventional oral health instruction by a dental hygienist	By subjects themselves with rear camera, DM scan box, and cheek retractors	Non-disclosed dental plaque	No reminder	Plaque index Plaque Control Record Gingival index Probing Depth Clinical Attachment Loss
**Snider et al^25^**	NRCT	Patients receiving orthodontic treatment (mean age: 24.8 years; mean age for male: 19.0 years; mean age for female: 30.0 years)	Total (n) = 49 Study group (n=24) using Dental Monitoring (DM) app; Control group (n=25)	Study group: monitored weekly by DM app during the treatment process; weekly personalised notifications from the app for 13 months Control group: received no additional monitoring during the treatment process	By subjects themselves with rear camera, DM scan box, and cheek retractors	Disclosed dental plaque	No reminder	Orthodontic Plaque index Modified gingival index

NRCT = non-randomised comparative study; RCT = randomised comparative study.

### Features of OHAI Advisor applications

Two applications (apps) have been developed and used for OHAI Advisors. Dental Monitoring (DM) was reported to be able to monitor disclosed or non-disclosed dental plaque and periodontal health,^20^^,^^24^^,^^25^ while WhiteTeeth was reported to detect disclosed dental plaque only.^21^ DM provides specific instructions on taking the intraoral selfie using the cheek retractor and scan box accessories.^20^^,^^24^^,^^25^ Training on how to take selfies was provided in each study.^20^^,^^24^^,^^25^ The study using WhiteTeeth^21^ did not offer specific instructions on selfie-taking. In all included studies,^20^^,^^21^^,^^24^^,^^25^ the AI-powered applications could generate an automatic response or notification upon detecting the oral hygiene status of the intraoral selfies. One study^24^ added professional human consultation following the automated AI response. No study specified smartphone or camera specifications. Three studies with the DM app^20^^,^^24^^,^^25^ used the smartphone’s rear camera, while WhiteTeeth^21^ only mentioned using a selfie. Two studies^21^^,^^25^ instructed subjects to apply a disclosing agent to their teeth before using the OHAI Advisor systems. In three studies,^20^^,^^24^^,^^25^ no explicit mention was made of regular reminders for subjects to use the OHAI Advisor systems. In contrast, one study^21^ mentioned that the app automatically sent periodic reminder messages.

### Study Designs

In two studies,^20^^,^^25^ both the study group and the control group were instructed at baseline to brush their teeth at least twice a day and floss once a day. However, one of these studies^20^ provided additional dietary advice. Both studies^20^^,^^25^ monitored the study groups with OHAI Advisor systems throughout the study period.

In one study,^21^ the study group received oral health content from the OHAI Advisor system, while the control group received “routine oral health education” and oral health instructions. However, there were no reports on specific details of the content delivered or how it was provided.

In another study,^24^ both the study group and the control group at baseline received 30-min one-on-one oral hygiene instructions (OHI) from a dental hygienist. These instructions^24^ covered periodontitis-related knowledge, toothbrushing techniques, interdental cleaning skills, and a demonstration of oral hygiene practices using disclosing agents. After that, the study group received AI-assisted real-person OHI based on routine intraoral selfies, while the control group only received real-person OHI.^24^

### Clinical Outcomes

The reported clinical outcomes are summarised in Table 3. The plaque index (PI) and gingival index (GI) were the most common measurement tools used to assess the clinical improvement of oral health in the included studies. Three studies^20^^,^^21^^,^^24^ showed decreasing PIs and GIs over the follow-up period for the study and control groups compared to their respective baseline scores, except for Snider et al,^25^ which showed increasing scores throughout the 13-month study period, indicating worsening oral hygiene.

**Table 3:** Reported clinical outcomes over time from the included studies

Outcomes	Study (by alphabetical order)	Groups	Baseline	1M	2M	3M		4M	5M	6M	7M	8M	9M	10M	11M	12M	13M
Mean Plaque Index	Sangalli et al^20^	Study	0.51	0.41	0.42	0.35#		-	-	0.31*#	-	-	-	-	-	-	-
		Control	0.44	0.63	0.69	0.74#		-	-	0.56*#	-	-	-	-	-	-	-
	Scheerman et al^21^ (Al-Anezi and Harradine PI)	Study	70.79	52.41	-	54.62*		-	-	-	-	-	-	-	-	-	-
		Control	75.34	62.97	-	70.42*		-	-	-	-	-	-	-	-	-	-
	Shen et al^24^	Study (AI)	1.1	0.8	-	0.6*##		-	-	-	-	-	-	-	-	-	-
		Study (AI+Human)	1.3	0.8#	-	0.6*##		-	-	-	-	-	-	-	-	-	-
		Control	1.2	1.0#	-	1.1#		-	-	-	-	-	-	-	-	-	-
	Snider et al^25^ (Orthodontic PI)	Study	0.32#	1.50	1.83	1.93		2.05	2.00#	1.5-2.0	>2.0	2.0-2.5	2.0-2.5	2.0-2.5	2.0-2.5	2.0-2.5	2.0-2.5
		Control	0.75#	1.53	1.90	2.17		2.19	2.75#	-	-	-	-	-	-	-	-
Mean Gingival Index	Sangalli et al^20^	Study	0.88#	0.61	0.45	0.43		-	-	0.36*	-	-	-	-	-	-	-
		Control	0.43#	0.48	0.58	0.56		-	-	0.47*	-	-	-	-	-	-	-
	Shen et al^24^	Study (AI)	1.2	1.0	-	0.8*#		-	-	-	-	-	-	-	-	-	-
		Study (AI+Human)	1.3	0.8*	-	0.7*#		-	-	-	-	-	-	-	-	-	-
		Control	1.4	1.2	-	1.0*		-	-	-	-	-	-	-	-	-	-
	Snider et al^25^ (Modified GI)	Study	0.59#	1.28	1.07#	1.46		1.51	1.60#	<1.5	1.5-2.0	1.5-2.0	2.0-2.5	>2.0	2.0-2.5	2.0-2.5	2.0-2.5
		Control	1.01#	1.45	1.85#	2.02		1.90	2.63#	-	-	-	-	-	-	-	-
Mean Probing Depth	Shen et al^24^	Study (AI)	9.0	-	-	7.7*#		-	-	-	-	-	-	-	-	-	-
		Study (AI+Human)	8.6	-	-	6.7*#		-	-	-	-	-	-	-	-	-	-
		Control	8.7	-	-	8.2*#		-	-	-	-	-	-	-	-	-	-
Clinical Attachment Loss	Shen et al^24^	Study (AI)	9.3	-	-	7.8*#		-	-	-	-	-	-	-	-	-	-
		Study (AI+Human)	8.9	-	-	6.8*#		-	-	-	-	-	-	-	-	-	-
		Control	9.1	-	-	8.4*#		-	-	-	-	-	-	-	-	-	-
Plaque Control Record	Shen et al^24^	Study (AI)	67.8	52.3*	-	47.2*#		-	-	-	-	-	-	-	-	-	-
		Study (AI+Human)	74.4	54.3*#	-	38.9*#		-	-	-	-	-	-	-	-	-	-
		Control	68.5	59.0*#	-	57.3*#		-	-	-	-	-	-	-	-	-	-
Bleeding on Marginal Probing Index	Scheerman et al^21^	Study	27.81	23.46	-	24.61		-	-	-	-	-	-	-	-	-	-
		Control	28.11	26.48*	-	27.63		-	-	-	-	-	-	-	-	-	-
White Spot Lesion	Sangalli et al^20^	Study	1.10	1.10	0.73	0.66		-	-	0.80*	-	-	-	-	-	-	-
		Control	1.13	0.93	1.00	0.71		-	-	0.93	-	-	-	-	-	-	-

* Statistically significant difference from baseline values in each respective group. # Statistically significant difference between two respective groups of the symbol reported.

However, using OHAI Advisors statistically significantly improved PI and GI in three studies^20^^,^^21^^,^^24^ compared with the control group. Moreover, Shen et al^24^ reported that OHAI Advisor could statistically significantly improve plaque control, probing depth (PD), and clinical attachment loss (CAL) compared to both the baseline and the control groups. The study^24^ also reported that using OHAI Advisor with verbal counseling statistically significantly improved PI, GI, PD, and Plaque Control Record (PCR) compared to using OHAI Advisor alone.

### Participant-reported Outcomes

Only one study^21^ mentioned participant-reported outcomes related to OHAI Advisor, which found no statistically significant improvement in the oral health behaviour score and hygiene practice behaviours.

### Assessment of the Quality of the Included Studies

The quality assessment of studies is summarised in Table 4. Two non-randomised trials^20^^,^^25^ were deemed of poor quality due to high drop-out rates and poorly controlled group allocation or sampling procedures. In one of these studies,^20^ there was no report on the randomisation of participants, no mention of blinding, and no mention of testing for baseline balance. Additionally, there were no details on drop-out rates. The other study^25^ also had problems with randomisation and blinding, although a test for baseline balance was reported. Moreover, the study^25^ suffered from a high drop-out rate (>20%).

**Table 4:** Summary of quality assessment of the included studies

Study (in ­alphabetical order)	Q1	Q2	Q3	Q4	Q5	Q6	Q7	Q8	Q9	Q10	Q11	Q12	Q13	Q14	Total score
Sangalli et al^20^	N	NR	N	N	N	N	NA	NA	Y	Y	Y	Y	Y	NR	5/14
Scheerman et al^21^	Y	Y	Y	Y	Y	Y	Y	Y	Y	Y	Y	Y	Y	Y	14/14
Shen et al^24^	Y	Y	Y	N	N	Y	N	N	Y	Y	Y	Y	Y	Y	10/14
Snider et al^25^	N	NR	NA	N	N	Y	N	N	Y	Y	Y	Y	Y	NR	6/14

One randomised controlled trial^24^ was considered to be of fair quality, with most domains well controlled, including randomisation, testing for baseline balance, and reported high adherence to the intervention protocols. However, neither the participants nor the assessors were blinded, and the study^24^ also had a high drop-out rate (>20%).

The remaining RCT study^21^ was deemed of good quality, with all domains well controlled, including randomisation, blinding, testing for baseline balance, low drop-out rates, and adherence to the intervention protocols. Detailed assessments for each study are presented in the appendix.

## DISCUSSION

The review’s findings highlight the recent and increased use of OHAI Advisors in dentistry, and support the hypothesis regarding the short-term effectiveness of OHAI Advisors in promoting oral health. Three included studies, Sangalli et al,^20^ Scheerman et al,^21^ and Shen et al,^24^ demonstrated a statistically significant reduction in the plaque index at 3 months, 6 months, and 12 weeks post-intervention, respectively, compared to the baseline. The gingival index also statistically significantly decreased in the investigations by Sangalli et al^20^ and Shen et al.^24^ Moreover, Shen et al^24^ reported improvements in plaque control, probing depth, and clinical attachment level in non-surgical periodontal treatments (NSPT) administered by one periodontist including full-mouth scaling, root planning, and OHI using OHAI Advisor. These investigations demonstrated that OHAI Advisors were superior to conventional face-to-face OHI in controlling oral hygiene in the short term, as evidenced by statistically significantly different clinical results.

For a longer-term effect of OHAI Advisors on oral health, a study with 13 months of follow-up by Snider et al^25^ reported a deterioration in oral hygiene throughout orthodontic treatment, regardless of whether OHAI Advisor was used or not. This was indicated by increases in both PI and GI. The authors hypothesised that motivation and willingness to scan may influence long-term practice.^25^ More studies are needed to investigate the long-term effectiveness of promoting oral health using OHAI advisors.

Shen et al^24^ reported that combining OHAI Advisor with human counseling was more effective than using OHAI Advisor alone. This suggests that regular follow-ups with healthcare professionals might still be beneficial. mHealth enables bi-directional communication between healthcare professionals and patients, facilitating disease monitoring and simple intervention delivery.^27^ However, messages delivered by AI systems were perceived as repetitive and impersonal, highlighting the need for personalised oral health education for both AI-powered and human-delivered approaches.^24^ With more generative AI (GenAI) technology advancements, the development of personalised AI-powered oral health education may be possible.^6^

Potential limitations of OHAI Advisors and the included investigations should be acknowledged. OHAI Advisors are designed to assess selfies; thus, the general population may assess only the labial and buccal surfaces of anterior teeth. The lingual surfaces and/or most of the posterior teeth were usually not assessed or were difficult to assess by the general population.^24^ The varying resolution of different smartphone cameras could affect the quality of intraoral photographs. Using front and rear smartphone cameras may also affect the resolution and, therefore, the quality of photographs. Furthermore, the number of OHAI Advisor apps and the types of study participants are limited; only two apps have been studied, and most participants were adolescents receiving fixed orthodontic treatments.

The Hawthorne effect,^16^ an improvement in performance due to awareness of being observed,^13^ may have contributed to the short-term effectiveness of OHAI Advisors, particularly in patients receiving orthodontic treatment.^9^ The included studies observed a consistent drop in remaining participants at every follow-up. These limitations call for caution in extrapolating the effectiveness of OHAI Advisors for future preventive dentistry. Thus, high-quality clinical trials or cross-sectional investigations with long-term assessments are needed.

## CONCLUSIONS

Within its limitations, OHAI Advisors appear effective for short-term oral hygiene maintenance. Further studies are required to demonstrate long-term effectiveness and user adherence. Moreover, research should focus on improving technical aspects such as the accuracy of the AI systems, data collection methods, and user interface. The preventive capability of the OHAI Advisors should also be developed by modifying approaches and enhancing value with regular verbal communication to increase applicability in clinical practice and community care.

## Figures and Tables

**Table A1:** NHI quality assessment for controlled intervention studies of Sangali et al^20^

Criteria	Yes	No	Other (CD, NR, NA)*
1. Was the study described as randomised, a randomised trial, a randomised clinical trial, or an RCT?		N	
2. Was the method of randomisation adequate (i.e., use of randomly generated assignment)?			NR
3. Was the treatment allocation concealed (so that assignments could not be predicted)?		N	
4. Were study participants and providers blinded to treatment group assignment?		N	
5. Were the people assessing the outcomes blinded to the participants’ group assignments?		N	
6. Were the groups similar at baseline on important characteristics that could affect outcomes (e.g., demographics, risk factors, co-morbid conditions)?		N	
7. Was the overall drop-out rate from the study at endpoint 20% or lower of the number allocated to treatment?			NA
8. Was the differential drop-out rate (between treatment groups) at endpoint 15 percentage points or lower?			NA
9. Was there high adherence to the intervention protocols for each treatment group?	Y		
10. Were other interventions avoided or similar in the groups (e.g., similar background treatments)?	Y		
11. Were outcomes assessed using valid and reliable measures, implemented consistently across all study participants?	Y		
12. Did the authors report that the sample size was sufficiently large to be able to detect a difference in the main outcome between groups with at least 80% power?	Y		
13. Were outcomes reported or subgroups analysed prespecified (i.e., identified before analyses were conducted)?	Y		
14. Were all randomised participants analysed in the group to which they were originally assigned, i.e., did they use an intention-to-treat analysis?			NR
Overall evaluation	POOR		

**Table A2:** NHI quality assessment for controlled intervention studies of Scheerman et al^21^

Criteria	Yes	No	Other (CD, NR, NA)*
1. Was the study described as randomised, a randomised trial, a randomised clinical trial, or an RCT?	Y		
2. Was the method of randomisation adequate (i.e., use of randomly generated assignment)?	Y		
3. Was the treatment allocation concealed (so that assignments could not be predicted)?	Y		
4. Were study participants and providers blinded to treatment group assignment?	Y		
5. Were the people assessing the outcomes blinded to the participants’ group assignments?	Y		
6. Were the groups similar at baseline on important characteristics that could affect outcomes (e.g., demographics, risk factors, co-morbid conditions)?	Y		
7. Was the overall drop-out rate from the study at endpoint 20% or lower of the number allocated to treatment?	Y		
8. Was the differential drop-out rate (between treatment groups) at endpoint 15 percentage points or lower?	Y		
9. Was there high adherence to the intervention protocols for each treatment group?	Y		
10. Were other interventions avoided or similar in the groups (e.g., similar background treatments)?	Y		
11. Were outcomes assessed using valid and reliable measures, implemented consistently across all study participants?	Y		
12. Did the authors report that the sample size was sufficiently large to be able to detect a difference in the main outcome between groups with at least 80% power?	Y		(Refer to their published protocol in which it is described)
13. Were outcomes reported or subgroups analysed prespecified (i.e., identified before analyses were conducted)?	Y		
14. Were all randomised participants analysed in the group to which they were originally assigned, i.e., did they use an intention-to-treat analysis?	Y		
Overall evaluation	GOOD		

**Table A3:** NHI quality assessment for controlled intervention studies of Shen et al^24^

Criteria	Yes	No	Other (CD, NR, NA)*
1. Was the study described as randomised, a randomised trial, a randomised clinical trial, or an RCT?	Y		
2. Was the method of randomisation adequate (i.e., use of randomly generated assignment)?	Y		
3. Was the treatment allocation concealed (so that assignments could not be predicted)?	Y		
4. Were study participants and providers blinded to treatment group assignment?		N	
5. Were the people assessing the outcomes blinded to the participants’ group assignments?		N	
6. Were the groups similar at baseline on important characteristics that could affect outcomes (e.g., demographics, risk factors, co-morbid conditions)?	Y		
7. Was the overall drop-out rate from the study at endpoint 20% or lower of the number allocated to treatment?		N	
8. Was the differential drop-out rate (between treatment groups) at endpoint 15 percentage points or lower?		N	
9. Was there high adherence to the intervention protocols for each treatment group?	Y		
10. Were other interventions avoided or similar in the groups (e.g., similar background treatments)?	Y		
11. Were outcomes assessed using valid and reliable measures, implemented consistently across all study participants?	Y		
12. Did the authors report that the sample size was sufficiently large to be able to detect a difference in the main outcome between groups with at least 80% power?	Y		
13. Were outcomes reported or subgroups analysed prespecified (i.e., identified before analyses were conducted)?	Y		
14. Were all randomised participants analysed in the group to which they were originally assigned, i.e., did they use an intention-to-treat analysis?	Y		
Overall evaluation	FAIR		

**Table A4:** NHI quality assessment for controlled intervention studies of Snider et al^25^

Criteria	Yes	No	Other (CD, NR, NA)*
1. Was the study described as randomised, a randomised trial, a randomised clinical trial, or an RCT?		N	
2. Was the method of randomisation adequate (i.e., use of randomly generated assignment)?			NR
3. Was the treatment allocation concealed (so that assignments could not be predicted)?			NA
4. Were study participants and providers blinded to treatment group assignment?		N	
5. Were the people assessing the outcomes blinded to the participants’ group assignments?		N	
6. Were the groups similar at baseline on important characteristics that could affect outcomes (e.g., demographics, risk factors, co-morbid conditions)?	Y		
7. Was the overall drop-out rate from the study at endpoint 20% or lower of the number allocated to treatment?		N	
8. Was the differential drop-out rate (between treatment groups) at endpoint 15 percentage points or lower?		N	
9. Was there high adherence to the intervention protocols for each treatment group?	Y		
10. Were other interventions avoided or similar in the groups (e.g., similar background treatments)?	Y		
11. Were outcomes assessed using valid and reliable measures, implemented consistently across all study participants?	Y		
12. Did the authors report that the sample size was sufficiently large to be able to detect a difference in the main outcome between groups with at least 80% power?	Y		
13. Were outcomes reported or subgroups analysed prespecified (i.e., identified before analyses were conducted)?	Y		
14. Were all randomised participants analysed in the group to which they were originally assigned, i.e., did they use an intention-to-treat analysis?			NR
Overall evaluation	POOR		
